# Supplementary figures and images for: Phenology and ecological role of aerobic anoxygenic phototrophs in freshwaters
Source: Microbiome. 2024 Mar 27;12:65. doi: 10.1186/s40168-024-01786-0 (PMC10976687; doi:10.1186/s40168-024-01786-0)

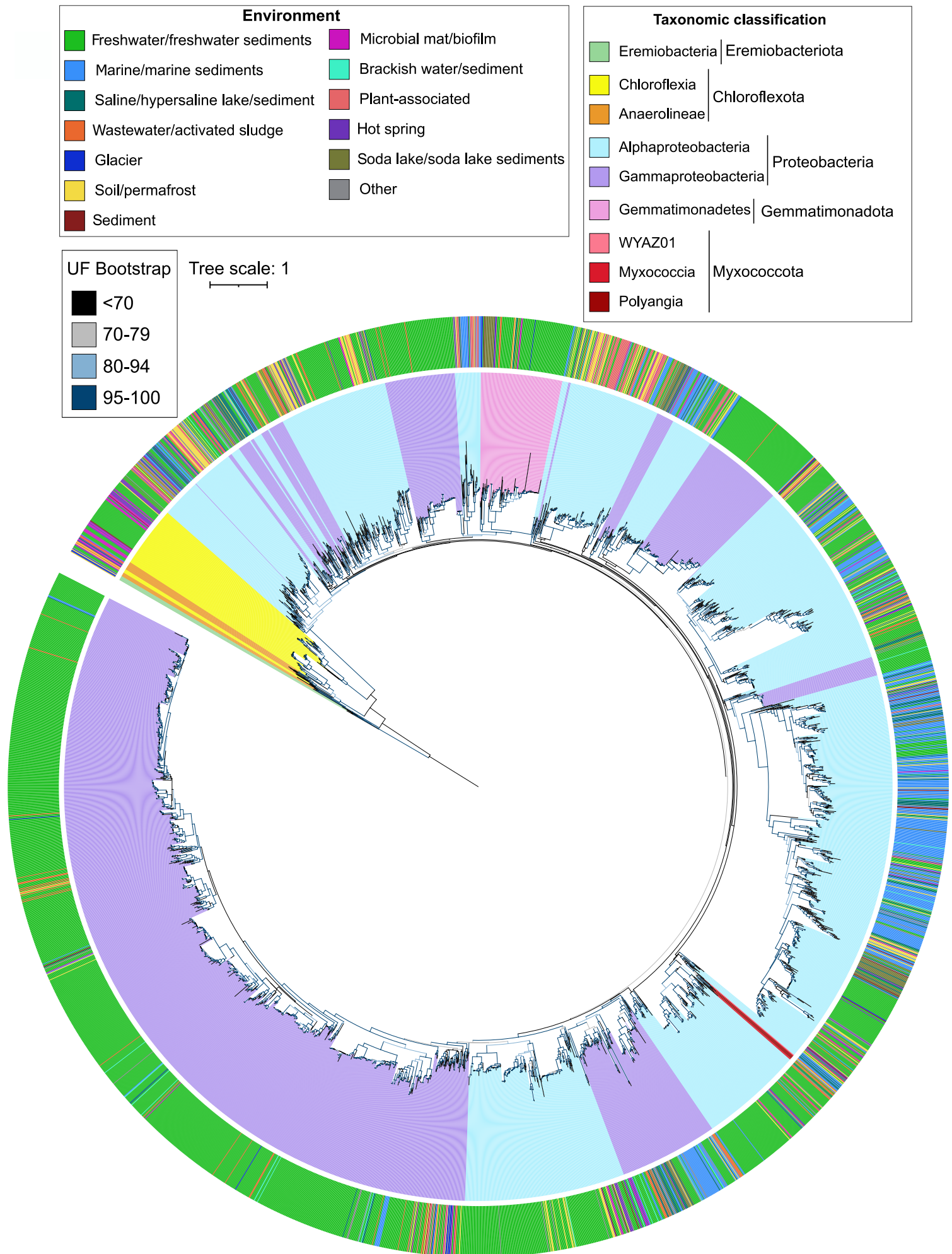

Supplement: Supplementary file 1 — Additional file 1: Supplementary Figure S1. Maximum likelihood phylogenetic tree of pufM gene sequences of the constructed database. Outer ring represents the environment of origin and the colour of the clades between branches and the outer ring shows the taxonomic classification of the sequences at class level. Colour of the branches refers to the ultra-fast bootstrap values. [file 40168_2024_1786_MOESM1_ESM.pdf]
