# Supplementary material for: Phenology and ecological role of aerobic anoxygenic phototrophs in freshwaters
Source: Microbiome. 2024 Mar 27;12:65. doi: 10.1186/s40168-024-01786-0 (PMC10976687; doi:10.1186/s40168-024-01786-0)
Supplement: Supplementary file 2 — Additional file 2: Supplementary Figure S2. Alphaproteobacteria community composition at order and genus level for 3-year sampling at 0.5 (A), 2 (B), 5 (C) and 8 m depth (D). Larger size and brighter colours are directly proportional to the relative contribution of each genus to the total Alphaproteobacteria community. [file 40168_2024_1786_MOESM2_ESM.pdf]

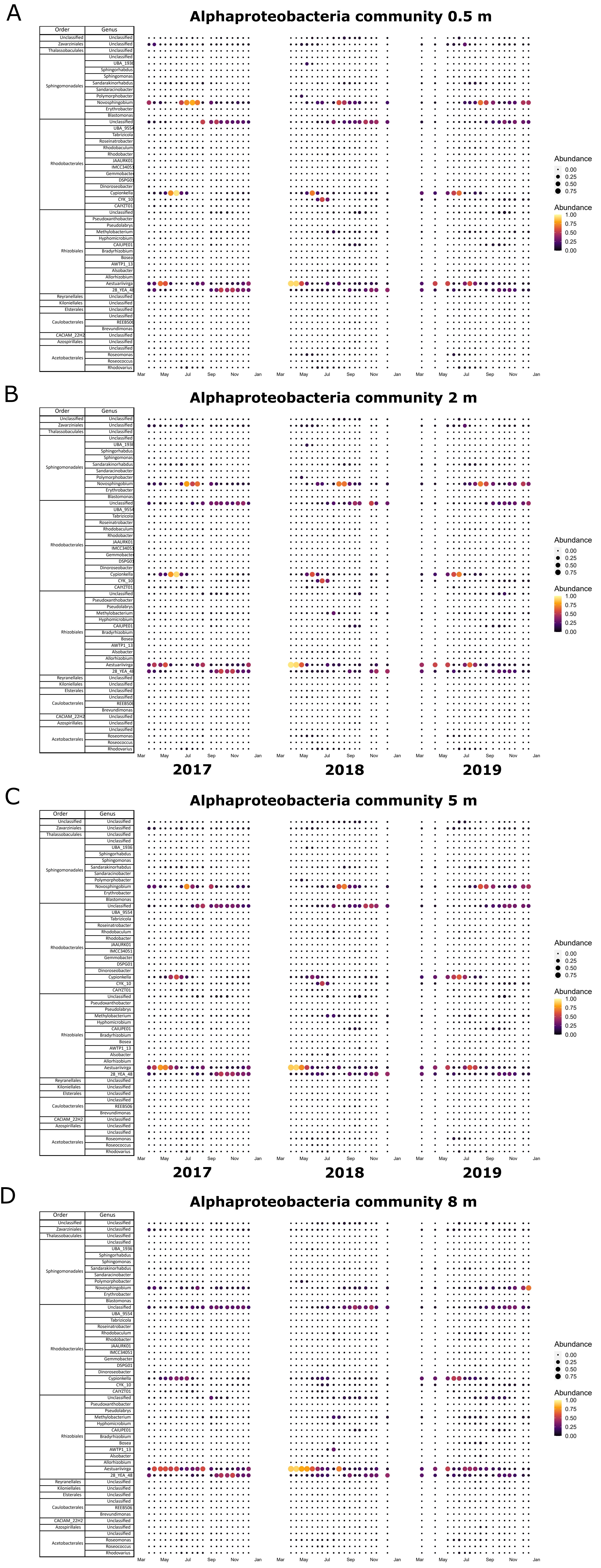

Supplementary Figure S2: Alphaproteobacteria community composition at order and genus level for 3-year sampling at 0,5 (A), 2 (B), 5 (C) and 8 m depth (D). Larger size and brighter colours are directly proportional to the relative contribution of each genus to the total Alphaproteobacteria community.
