# Supplementary material for: Phenology and ecological role of aerobic anoxygenic phototrophs in freshwaters
Source: Microbiome. 2024 Mar 27;12:65. doi: 10.1186/s40168-024-01786-0 (PMC10976687; doi:10.1186/s40168-024-01786-0)
Supplement: Supplementary file 5 — Additional file 5: Supplementary Figure S5. Environmental and biological variables for 8 m’ depth profile during 3-year sampling in CEP lake. Temperature (A), AAP abundance (B), dissolved oxygen (C), percentage contribution to total bacterial community (D), Shannon alpha diversity values (E), and Chlorophyll-a (F). Light-blue vertical bands represent lack of sampling due to frozen lake surface. [file 40168_2024_1786_MOESM5_ESM.pdf]

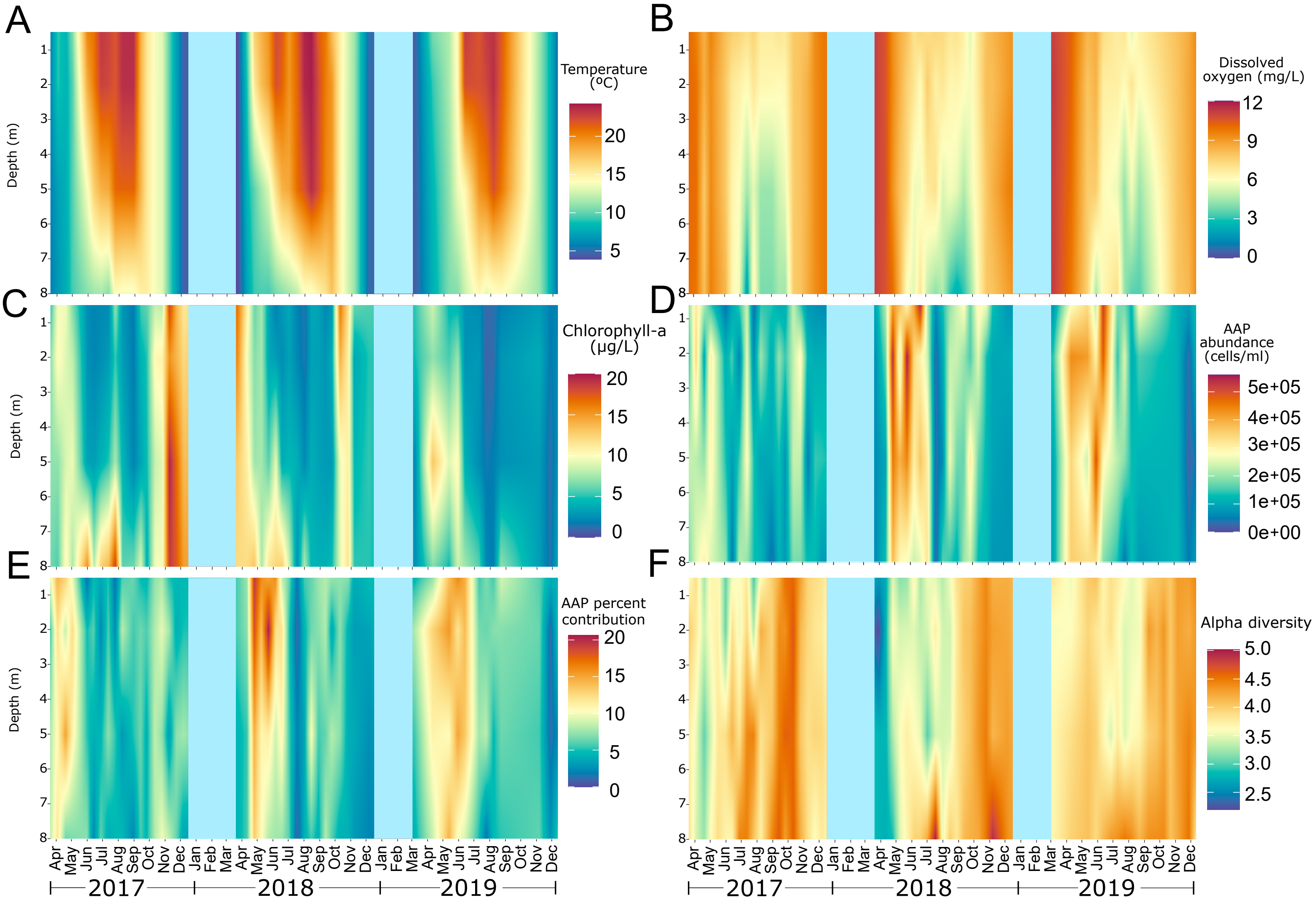

Supplementary Figure S5: Environmental and biological variables for 8 meters' depth profile during 3-year sampling in CEP lake. Temperature (A), AAP abundance (B), dissolved oxygen (C), percentage contribution to total bacterial community (D), Shannon alpha diversity values (E), and Chlorophyll-*a* (F). Light-blue vertical bands represent lack of sampling due to frozen lake surface.
