# Supplementary material for: Phenology and ecological role of aerobic anoxygenic phototrophs in freshwaters
Source: Microbiome. 2024 Mar 27;12:65. doi: 10.1186/s40168-024-01786-0 (PMC10976687; doi:10.1186/s40168-024-01786-0)
Supplement: Supplementary file 6 — Additional file 6: Supplementary Figure S6. Phytoplankton chloroplast-based community composition at class level for 0.5, 2, 5 and 8 m’ depth during 3-years temporal series. [file 40168_2024_1786_MOESM6_ESM.pdf]

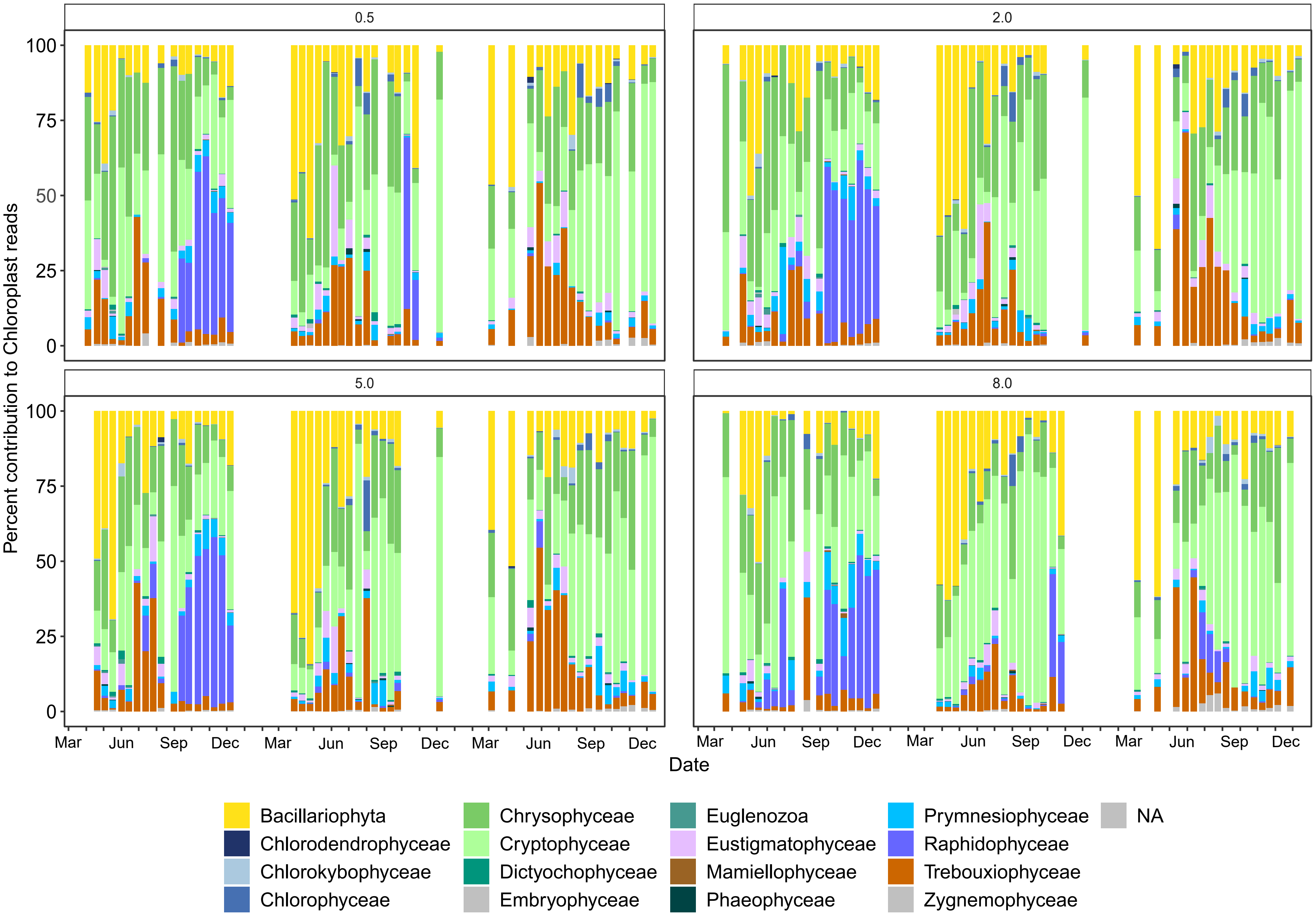

Supplementary Figure S6: Phytoplankton chloroplast-based community composition at class level for 0.5, 2, 5 and 8 meters' depth during 3-years temporal series.
