# Supplementary material for: Phenology and ecological role of aerobic anoxygenic phototrophs in freshwaters
Source: Microbiome. 2024 Mar 27;12:65. doi: 10.1186/s40168-024-01786-0 (PMC10976687; doi:10.1186/s40168-024-01786-0)
Supplement: Supplementary file 7 — Additional file 7:Supplementary Figure S7. Decomposition of additive time series for bacterial abundance (a), AAP abundance (b), chlorophyll-a concentration (c) and temperature (d). Analysis was done in the TTR package version 0.24.3 (R v4.3.0). Spearman correlation of the decomposed trends between AAP abundance and chlorophyll-a (e) and AAP abundance and temperature (f). R: spearman’s rho value, p: p-value. [file 40168_2024_1786_MOESM7_ESM.pdf]

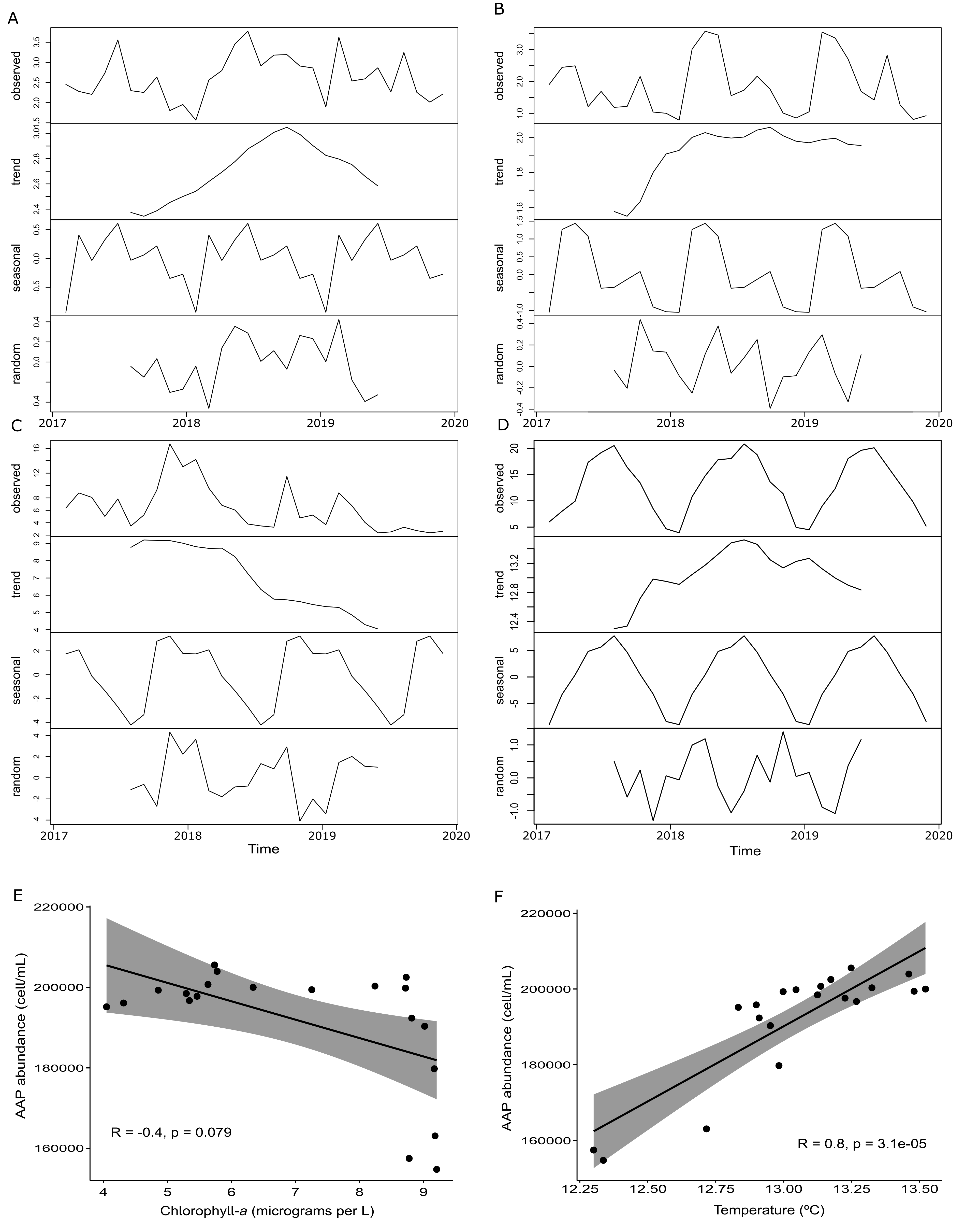

<u>Supplementary Figure S7</u> : Decomposition of additive time series for bacterial abundance (A), AAP abundance (B), chlorophyll -*a* concentration (C) and temperature (D). Analysis was done in the TTR package version 0.24.3 (R version 4.3.0). Spearman correlation of the decomposed trends between AAP abundance and chlorophyll-a (E) and AAP abundance and temperature (F). R: spearman's rho value, p: p-value.
