# Supplementary material for: Phenology and ecological role of aerobic anoxygenic phototrophs in freshwaters
Source: Microbiome. 2024 Mar 27;12:65. doi: 10.1186/s40168-024-01786-0 (PMC10976687; doi:10.1186/s40168-024-01786-0)
Supplement: Supplementary file 8 — Additional file 8: Supplementary Figure S8. AAP bacteria community composition according to pufM gene taxonomic assignment at class level for 0.5 (A), 2 (B), 5 (C) and 8 meters’ depth (D) during 3-years sampling campaign. [file 40168_2024_1786_MOESM8_ESM.pdf]

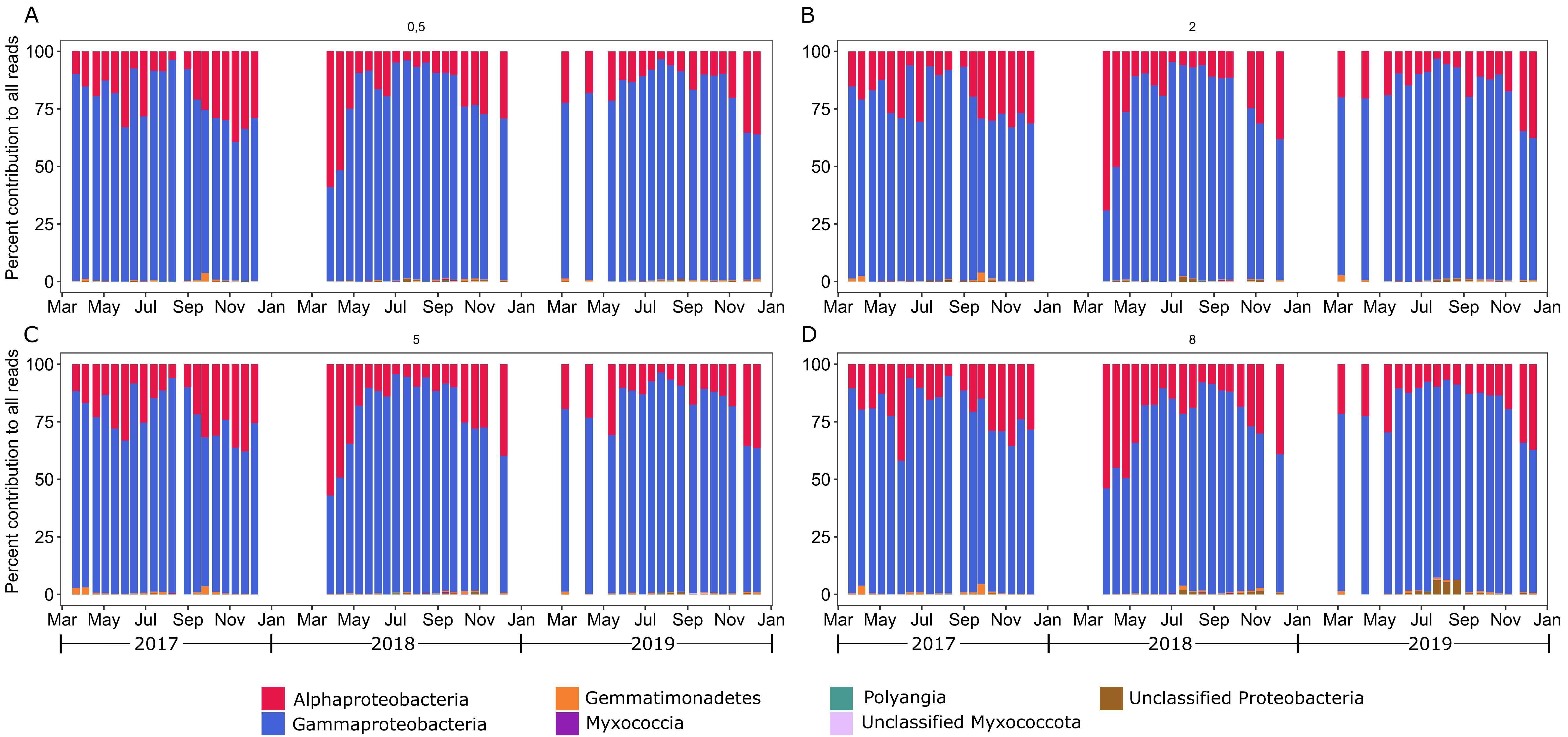

<u>Supplementary Figure S8</u>: AAP bacteria community composition according to *puf*M gene taxonomic assignment at class level for 0.5 (A), 2 (B), 5 (C) and 8 meters' depth (D) during 3-years sampling campaign.
