# Supplementary material for: Phenology and ecological role of aerobic anoxygenic phototrophs in freshwaters
Source: Microbiome. 2024 Mar 27;12:65. doi: 10.1186/s40168-024-01786-0 (PMC10976687; doi:10.1186/s40168-024-01786-0)
Supplement: Supplementary file 9 — Additional file 9: Supplementary Figure S9. Individually normalized relative abundance of the 22 core AAP ASVs during 3 years in 4 depths. Brighter colours and bigger dots indicate larger contribution to the AAP bacterial community. ASVs are clustered according to taxonomic classification at the maximum possible level (genus, family or order). [file 40168_2024_1786_MOESM9_ESM.pdf]

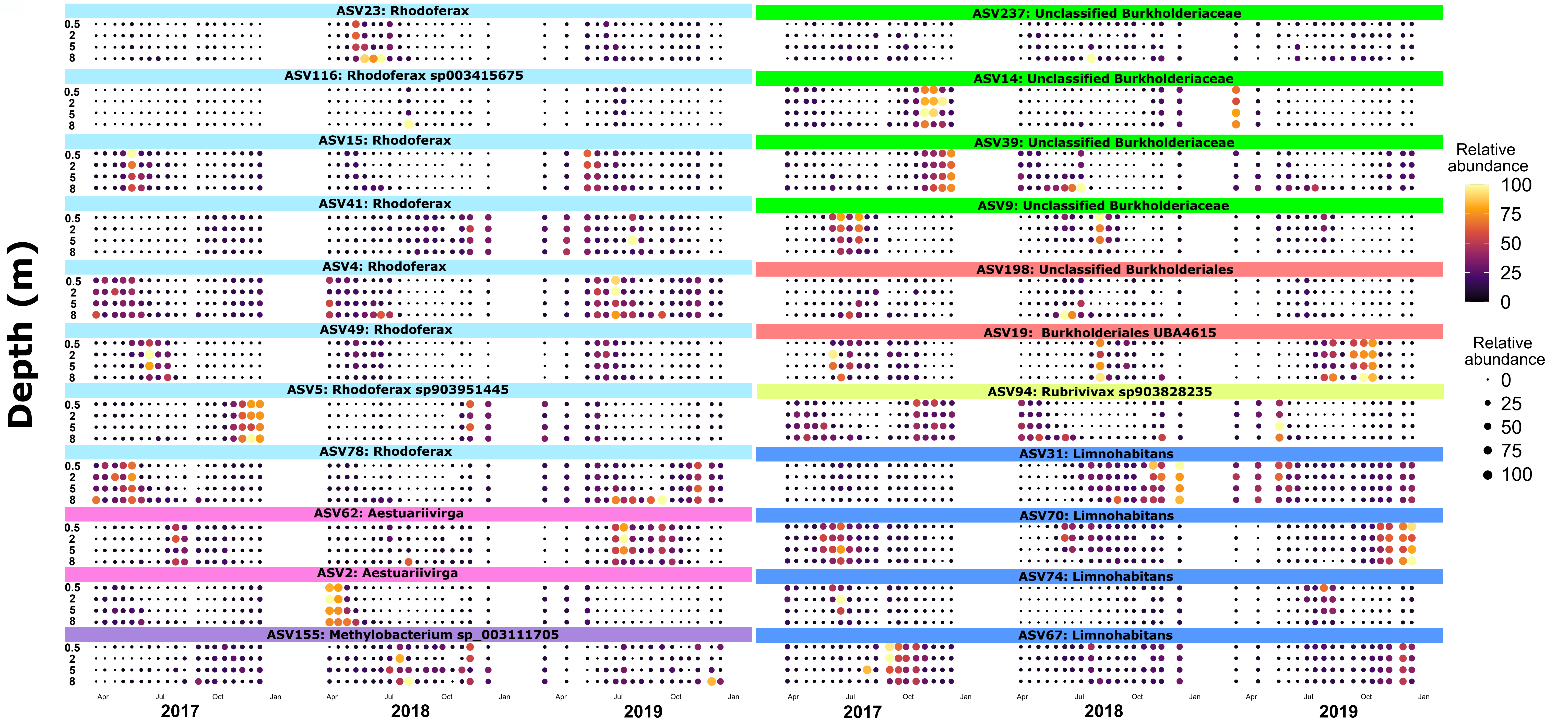

<u>Supplementary Figure S9</u>: Individually normalized relative abundance of the 22 core AAP ASVs during 3 years in 4 depths. Brighter colors and bigger dots indicate larger contribution to the AAP bacterial community. ASVs are clustered according to taxonomic classification at the maximum possible level (genus, family or order).
